# P279: Infection prevention and control program in developing countries: achievements, challenges and opportunities in Cambodia

**DOI:** 10.1186/2047-2994-2-S1-P279

**Published:** 2013-06-20

**Authors:** S Sok, Koum Kanal

**Affiliations:** 1 Department of Hospital Services, Ministry of Health, Phnom Penh, Cambodia

## Introduction

Over the last few years MoH and WHO have refocused their efforts on improving infection prevention and control (IPC) practice.

## Objectives

We describe here a cross departmental approach to improving IPC capacity.

## Methods

Initially we focused on developing national guidelines; however, we noticed absence of policy and strategy hindered IPC activities. Consequently, while developing guidelines, we worked with partners and other departments within MoH to develop national policy and strategic plan for IPC.

## Results

### Achievements

As a consequence of IPC policy and plan, we created a dedicated team at MoH, Provincial Health Departments (PHD), and hospitals. There are national guidelines and training curriculum. A cohort of trainers from national level and provincial hospitals is training hospital staff using the curriculum. Hospital facilities are being upgraded and supplies for infection control practice are added into essential drug list of MoH. We have piloted nosocomial infection surveillance and developing IPC model practice sites.

### Challenges

IPC continues to suffer from poor staff capacity and commitment, limited support by decision makers and financial shortages. Its cross cutting nature means that programs assume IPC is covered by someone else. Staff behavior change is one of the biggest challenges.

### Opportunities

Having IPC policy and plan gives us a tool for fund raising and advocacy. To ensure sustainability, the program is linking IPC with quality of care, using standard assessment tools to evaluate activities which will be used to provide dedicated funding. With approval from decision makers, the IPC team of MoH has been coordinating and guiding IPC teams in most provincial hospitals and PHDs to add budget for IPC activities into their annual operational plan.

## Conclusion

We moved IPC practice from a number of disjointed vertical programs to a cohesive, comprehensive mission. Next challenge is ensuring that staff practice IPC.

The evolution of IPC program in Cambodia may serve as a model for other countries that seek to initiate similar programs.

## Competing interests

None declared
